# Environmental Exposure to Polychlorinated Biphenyls and *p,p*´-DDE and Sperm Sex-Chromosome Disomy

**DOI:** 10.1289/ehp.1104017

**Published:** 2011-12-21

**Authors:** Megan E. McAuliffe, Paige L. Williams, Susan A. Korrick, Larisa M. Altshul, Melissa J. Perry

**Affiliations:** 1 Department of Environmental Health, and; 2 Department of Biostatistics, Harvard School of Public Health, Boston, Massachusetts, USA; 3 Channing Laboratory, Department of Medicine, Brigham and Women’s Hospital, Harvard Medical School, Boston, Massachusetts, USA; 4 Environmental Health and Engineering Inc., Needham, Massachusetts, USA; 5 Department of Environmental and Occupational Health, George Washington University School of Public Health and Health Services, Washington, DC, USA

**Keywords:** aneuploidy, dichlorodiphenyltrichloroethane (DDT), dichlorodiphenyldichloroethylene (DDE), disomy, endocrine disruptors, *in situ* hybridization, fluorescence, pesticides, polychlorinated biphenyls (PCBs), reproduction, sperm

## Abstract

Background: Chromosomal abnormalities contribute substantially to reproductive problems, but the role of environmental risk factors has received little attention.

Objectives: We evaluated the association of polychlorinated biphenyl (PCB) and dichlorodiphenyldichloroethylene (*p,p*´-DDE) exposures with sperm sex-chromosome disomy.

Methods: We conducted a cross-sectional study of 192 men from subfertile couples. We used multiprobe fluorescence *in situ* hybridization (FISH) for chromosomes X, Y, and 18 to determine XX, YY, XY, and total sex-chromosome disomy in sperm nuclei. Serum was analyzed for concentrations of 57 PCB congeners and *p,p*´-DDE. Poisson regression models were used to calculate incidence rate ratios (IRRs) for disomy by exposure quartiles, controlling for demographic characteristics and semen parameters.

Results: The median percent disomy was 0.3 for XX and YY, 0.9 for XY, and 1.6 for total sex-chromosome disomy. We observed a significant trend of increasing IRRs for increasing quartiles of *p,p*´-DDE in XX, XY, and total sex-chromosome disomy, and a significant trend of increasing IRRs for increasing quartiles of PCBs for XY and total sex-chromosome disomy; however, there was a significant inverse association for XX disomy.

Conclusions: Our findings suggest that exposure to *p,p*´-DDE may be associated with increased rates of XX, XY, and total sex-chromosome disomy, whereas exposure to PCBs may be associated with increased rates of YY, XY, and total sex-chromosome disomy. In addition, we observed an inverse association between increased exposure to PCBs and XX disomy. Further work is needed to confirm these findings.

Chromosomal abnormalities are responsible for a substantial portion of reproductive problems. Unlike the standard indices of sperm quality, the consequence of successful fertilization with an aneuploid sperm are clear and serious, resulting in either congenital abnormalities or pregnancy loss. Sex-chromosome aneuploidies are the most common chromosomal error and are seen in at least 5% of clinical births, with some abnormalities not being realized until puberty (Hassold and Hunt 2001). Whereas maternal oocyte nondisjunction accounts for > 95% of autosomal aneuploidy, sex chromosomal aneuploidy is known to be of paternal origin about 80% of the time (Hassold et al. 1996; Hassold and Hunt 2001). Sex-chromosome aneuploidy is seen among otherwise normal mature sperm with a frequency between 0.13 and 1.20% (Egozcue et al. 1997); another study reported the average sex-chromosome disomy across 23 studies to be 0.26% (Templado et al. 2005). Many studies have demonstrated an even higher frequency in infertile men (Bernardini et al. 1998; Calogero et al. 2001; Shi and Martin 2001). Although the rate and consequence of sperm sex-chromosome abnormalities are considerable, possible environmental risk factors for sex-chromosome disomy have not been well investigated in epidemiologic studies (Wyrobek et al. 2005).

Much concern has been raised about organochlorine chemicals such as polychlorinated biphenyls (PCBs) and dichlorodiphenyltrichloroethane (DDT) because of studies showing that both PCBs and dichlorodiphenyldichloroethylene (*p,p*´-DDE), the most stable daughter compound of DDT, are found in most of the population (Longnecker et al. 1997). Serum levels of PCBs and *p,p*´-DDE reflect long-term exposure. Depending on the congener type, PCBs have half-lives of 1–10 years, and *p,p*´-DDE has a half-life of ≥ 10 years.

PCBs and *p,p*´-DDE are persistent, lipophilic, endocrine-disrupting organochlorines that readily penetrate the blood–testis barrier (Bush et al. 1986). PCBs and *p,p*´-DDE could act via a number of possible mechanisms to alter endocrine homeostasis, which is necessary for testicular function. For example, *in vitro* and animal models have demonstrated that PCBs can bind to estrogen receptors (Korach et al. 1988), increase gonadotropin-releasing hormone levels, or affect production and release of luteinizing hormone from the pituitary (Jansen et al. 1993). *p,p*´-DDE may affect spermatogenesis through its antiandrogenic activity (Kelce et al. 1995, 1997). In rodent models, changes in the endocrinologic environment of the testes have been shown to affect the rate of meiotic segregation errors (Mroz et al. 1999; Oppedisano et al. 2002). Furthermore, in epidemiologic studies, PCBs and *p,p*´-DDE have been associated with decreased semen quality, a key indicator of male reproductive health (Ayotte et al. 2001; Charlier and Foidart 2005; Dalvie et al. 2004; Hauser et al. 2003; Pant et al. 2004, 2007). Several other male reproductive toxicants, such as nonpersistent pesticides (e.g., herbicides, fungicides, organophosphates) (Perry 2008) and benzene (Xing et al. 2010), have been associated with sperm aneuploidy, but no published studies to date have investigated the relationship between organochlorines and human sperm aneuploidy.

The present study was designed to investigate whether environmental exposure to PCBs and *p,p*´-DDE are associated with altered frequency of sperm sex-chromosome aneuploidy in adult men. We selected our study population without specific exposure (e.g., no known occupational exposure) to these compounds, because even small associations may have large public health relevance given the widespread exposures to PCBs and *p,p*´-DDE in the general population.

## Materials and Methods

*Subjects.* Subjects were a subset of participants enrolled in a study assessing the impacts of environmental exposures on semen among 341 men. Men 20–54 years of age from couples seeking infertility evaluation from the Massachusetts General Hospital (MGH) Fertility Center between January 2000 and May 2003 were eligible for this parent study. Sixty-five percent of eligible men agreed to participate. Most men who declined participation cited lack of time on the day of the clinic visit as their reason for nonparticipation. Men were excluded from the parent study if they were presenting for postvasectomy semen analysis and/or receiving treatment for infertility (i.e., hormonal treatments). Each participant provided a semen and blood sample and completed a self-administered questionnaire that collected information on race/ethnicity, medical and fertility history, and lifestyle factors. Men were eligible for this substudy if they had an available semen sample for fluorescence *in situ* hybridization (FISH) analysis. Semen sample availability was based on whether an aliquot of sample was available for use from the biorepository, as this cohort has been used for other semen analysis research. There were no other additional eligibility requirements. Of the 341 men enrolled in the parent study, sufficient semen sample was available for 192 men (56%). The parent study was approved by the Harvard School of Public Health and MGH human subjects committees. All subjects signed an informed consent form prior to participation.

*Semen analysis.* All subjects were asked to abstain from ejaculation for 48 hr prior to giving their single semen sample by masturbation. Samples were liquefied at 37°C for 20 min before analysis. Analysis of fresh samples took place at the MGH Andrology Laboratory by andrologists blinded to exposure status. The volume, pH, color, and viscosity were determined for each fresh sample. Sperm counts and percent motility were first determined manually, then measured by computer-aided sperm analysis (CASA) using the Hamilton-Thorn Motility Analyzer (10HTM-IVO; Hamilton Thorne Inc., Beverly, MA). A minimum of 200 sperm from four different fields was analyzed. CASA provides data on rapid and linear motile sperm (percent), velocity of average path (micrometers per second), amplitude of lateral head displacement (micrometers), linearity, and other sperm movement characteristics. Additionally, each sample was prepared on two slides for morphological assessment using a Nikon microscope with an oil immersion 100× objective (Nikon Company, Tokyo, Japan). Sperm were scored as normal or abnormal using Kruger strict criteria (Kruger et al. 1988).

*Serum PCB and* p,p*´-DDE measurements.* On the same day as semen sample collection, nonfasting blood samples were collected, frozen at–80°C, and subsequently analyzed by the Organic Chemistry Analytical Laboratory at the Harvard School of Public Health. The target analytes were 57 individual PCB congeners and *p,p*´-DDE. Details of the sampling, analytical, and quality control procedures are published elsewhere (Hauser et al. 2003; Korrick et al. 2000). Briefly, serum extracts were analyzed using gas chromatography with electron capture detection. Quantification was based on the response factors of individual PCB congeners and *p,p*´-DDE relative to internal standards. PCB concentrations were reported as individual congeners.

To adjust for variation in serum lipid levels among the nonfasting blood samples (Phillips et al. 1989), we measured percent lipid gravimetrically for each sample by weighing an aliquot of sample extract evaporated to dryness. Method detection limits (MDLs) were determined as recommended by the U.S. Environmental Protection Agency (1984). The MDL values for all PCB congeners and *p,p*´-DDE were < 0.05 ng/g, and most congeners had MDLs < 0.01 ng/g.

*X-Y-18 sperm FISH.* One investigator (M.E.M.) performed FISH, imaging, and nuclei scoring analyses on frozen aliquots of all samples. She was blinded to exposure and covariate status while preparing and analyzing all samples. FISH was carried out for chromosomes X, Y, and 18 (autosomal control). Sex chromosomes were of primary interest, as they are most susceptible to disomy; however, 1818 disomy was also evaluated as a secondary outcome. Image processing and scoring were performed using custom software developed in MATLAB (Mathworks Inc., Natick, MA), using classification and scoring algorithms based on the scoring criteria described by Baumgartner et al. (1999). The overall hybridization efficiency was 97%, which is consistent with the hybridization efficiency reported by other groups (Johannisson et al. 2002; Martin et al. 1996; Tiido et al. 2005). These methods have been described in detail elsewhere (Perry et al. 2011).

*Statistical analysis.* Descriptive statistics for demographic characteristics and semen parameters were calculated. We used World Health Organization cutoffs (World Health Organization 1999) to categorize normal sperm concentration (≥ 20 million/mL), motility (≥ 50% motile), and morphology (≥ 4% normal). Because PCBs and *p,p*´-DDE were not normally distributed, nonparametric methods (Spearman correlation coefficients and Wilcoxon rank-sum tests) were used to explore their relationships with age, smoking status, abstinence time, race, and sperm concentration and motility.

We evaluated the relationship of sperm aneuploidy with *p,p*´-DDE exposure and with the sum of the four most prevalent individual PCB congeners (118, 138, 153, and 180; Σ_4_PCBs). We also conducted exploratory analyses using other biologically relevant groupings of PCB congeners, as proposed by Wolff et al. (1997), considering those that are estrogen-like (congeners 44, 49, 52, 101, 187, 174, 177, and 157/201; Σestrogenic PCBs) and those that are dioxin-like (congeners 95/66, 74, 77/110, 105/141, 118, 156, 167, 128, 138, and 170; Σdioxin-like PCBs). We also considered multiple PCB groupings simultaneously, but it was difficult to distinguish their independent effects because of the high intercorrelation among PCB subgroups.

We used Poisson regression models to explore the associations between organochlorines and sperm sex-chromosome disomy. We fitted Poisson models using counts of each of the disomy types (XX18, YY18, XY18, total sex-chromosome disomy, and 1818) for each subject as the outcome variable, the natural logarithm of the number of sperm nuclei scored [log(nuclei)] as the offset variable, and organochlorine levels classified in quartiles as the exposure variable, adjusting for other relevant covariates. In Poisson regression, the offset variable allows for control of time/space variation in the denominator, which in this study is the number of nuclei scored per subject. Because we found evidence of overdispersion (i.e., the variance exceeded the Poisson mean), robust methods based on generalized estimating equations were used to estimate exposure effects. Because of possible bias in standardizing exposure measures by serum lipids (Schisterman et al. 2005), we adjusted for serum lipids as a covariate in our models. Incidence rate ratios (IRRs) and 95% confidence intervals (CIs) were calculated using the first (lowest) quartile as the reference group.

We used scatter plots to explore the relationships among covariates and PCBs and *p,p*´-DDE. Age, abstinence time, and smoking were included in all models based on *a priori* considerations about their likely associations with outcome or exposure (Blackwell and Zaneveld 1992; Hassan and Killick 2003; Vine 1996). Other potential confounding variables assessed were sperm concentration, motility, and morphology; race/ethnicity; and body mass index (BMI). Because both semen concentration and motility were highly skewed and residuals were not normally distributed, they were log-transformed prior to being included in models. Linearity after log transformation was verified with scatter plots. All potential confounding variables were included in initial models. Inclusion in final models was based on either *a priori* considerations, as mentioned above, or a change of at least 10% in the main exposure effect. We included *p,p*´-DDE in Σ_4_PCB models, and vice-versa, to control for the other potential exposure.

Covariates in the final Poisson regression models were age (continuous), sperm concentration and motility (log-transformed continuous), abstinence time (≤ 2 days, 3–4 days, ≥ 5 days), serum lipids (continuous), and smoking status (never vs. ever). Additional models were fit to assess the appropriateness of assuming a linear relationship for the remaining continuous and ordinal covariates. Linear trend was determined by modeling quartiles of exposure as an ordinal variable assigned values of 1–4. Two-sided *p*-values < 0.05 were considered significant. We used SAS software, (version 9.2; SAS Institute Inc., Cary, NC) for all data analyses.

## Results

Table 1 shows the demographic and semen parameter characteristics of the 192 study participants. Overall, participants had a mean (± SD) age of 35.4 ± 5.1 years and a mean (± SD) BMI of 28.1 ± 5.4 kg/m^2^. Most of the participants were white (85%), with 3% African American, and 4% Hispanic. Most men (75%) had never smoked, 15 (8%) were current smokers, and 33 (17%) were ex-smokers. Of the 192 men in our study, 21 (11%) had sperm concentrations < 20 million/mL, 84 men (44%) had < 50% motile sperm, and 35 men (18%) had < 4% normally shaped sperm. Participants in this study did not differ significantly with respect to their demographic or semen parameter characteristics compared with those in the full parent study cohort (*n* = 341) (data not shown).

**Table 1:** Demographic characteristics and semen parameters for a subset of men seeking infertility evaluation, January 2000–May 2003 (*n* = 192).

Characteristic/parameter	*n* (%)
Age (years)		35.4 ± 5.1*a*
BMI (kg/m^2^)		28.1 ± 5.4*a*
Race/ethnicity		
White		163 (84.9)
African American		5 (2.6)
Hispanic		8 (4.2)
Other		16 (8.3)
Abstinence time (days)*b *		
≤ 2		41 (21.5)
3–4		90 (47.1)
≥ 5		60 (31.4)
Smoking status*c *		
Never smoker		142 (74.7)
Ever smoker		48 (25.3)
Current smoker		15 (7.9)
Former smoker		33 (17.4)
Semen parameter		
Concentration < 20 million/mL		21 (10.9)
Motility < 50% motile		84 (43.8)
Morphology < 4% normal		35 (18.2)
**a**Mean ± SD.** b**Information is missing for 1 man. **c**Information is missing for 2 men.		

We scored a median of 5,895 sperm nuclei per subject (Table 2). The observed median percentages of disomy were as follows: XX18, 0.3%; YY18, 0.3%; XY18, 0.9%; 1818, 0.04%; and total sex-chromosome disomy, 1.6%.

**Table 2:** Number of sperm nuclei scored and percent disomy for a subset of men seeking infertility evaluation, January 2000–May 2003 (*n* = 192).

Mean ± SD	Median	25th percentile	75th percentile
Nuclei (*n*)		6,961 ± 4,644		5,895		3,248		9,960
1818 disomy		0.13 ± 0.24		0.04		0.00		0.15
XX18 disomy		0.41 ± 0.40		0.27		0.16		0.50
YY18 disomy		0.38 ± 0.33		0.30		0.19		0.46
XY18 disomy		1.11 ± 0.83		0.89		0.56		1.46
Total disomy*a*		1.90 ± 1.26		1.56		1.10		2.47
**a**Total sex-chromosome disomy = ∑(XX18 + YY18 + XY18).								

The distribution of wet-weight concentrations of *p,p*´-DDE and PCBs in serum and serum lipids is provided in Table 3. The median wet-weight concentrations of Σ_4_PCBs and *p,p*´-DDE were 0.53 ng/g (range, 0.16– 6.14 ng/g) and 0.97 ng/g (range, 0.23–30.3 ng/g), respectively. The median percent serum lipids was 0.5% (range, 0.13–1.17%). The median lipid-adjusted concentrations for Σ_4_PCBs and were 110 ng/g lipids (range, 28.9–ng/g lipids) and 198 ng/g lipids (range, 34.8–7,776 ng/g lipids), respectively (data not shown). Serum levels of individual PCB congeners were lower than serum levels of *p,p*´-DDE (data not shown), a finding that is consistent with other U.S.-based studies (Korrick et al. 2000). PCB and *p,p*´-DDE levels in the present study were comparable with the general U.S. population, as reported in the *Fourth National Report on Human Exposure to Environmental Chemicals* (Centers for Disease Control and Prevention 2009). Correlations among PCB measures, including the most prevalent individual congeners and congener groupings, were high (*r* = 0.6–0.9) (data not shown). However, correlations between *p,p*´-DDE and most PCB measures were more modest (*r* = 0.4).

**Table 3:** Distribution of *p,p*´-DDE and PCBs (ng/g serum) and serum lipids for a subset of men seeking infertility evaluation, January 2000–May 2003 (*n* = 192).

Percentile									Geometric mean
Minimum	10th	25th	50th	75th	90th	Maximum			
*p,p*´-DDE		0.23		0.41		0.61		0.97		1.68		2.59		30.32		1.11
∑_57_PCBs*a*		0.31		0.54		0.69		0.97		1.50		2.20		9.86		1.02
∑_4_PCBs*b*		0.16		0.29		0.38		0.53		0.81		1.18		6.14		0.55
∑Estrogenic PCBs*c*		0.02		0.03		0.05		0.07		0.11		0.17		0.61		0.07
∑Dioxin-like PCBs*d*		0.10		0.19		0.25		0.35		0.56		0.83		3.47		0.37
Serum lipids (%)		0.13		0.29		0.39		0.50		0.64		0.80		1.17		0.48
**a**Includes congeners 6, 8, 16, 18, 25, 26, 28, 31, 33, 37, 41, 44, 47, 49, 52, 60, 95/66, 70, 74, 77/110, 84, 87, 97, 99, 101, 105/141, 118, 128, 135, 136, 138, 146, 149, 151, 153, 156, 157/210, 167, 170, 171, 174, 177, 180, 183, 187, 189, 194, 195, 196/203, 199, 206, 209. **b**Includes congeners 118, 138, 153, and 180. **c**Includes congeners 44, 49, 52, 101, 187, 174, 177, 157/201 (Wolff et al. 1997). **d**Includes congeners 95/66, 74, 77/110, 105/141, 118, 156, 167, 128, 138, 170 (Wolff et al. 1997).																

After adjusting for smoking status, abstinence time, sperm concentration and motility, and serum lipids, we observed that a 10-year increase in age was associated with increased rates of XX (IRR, 1.16; 95% CI: 1.07, 1.21) and YY disomy (IRR, 1.18; 95% CI: 1.10, 1.25) but was inversely associated with XY (IRR, 0.83; 95% CI: 0.71, 0.92) and total sex-chromosome disomy (IRR, 0.95; 95% CI: 0.88, 0.98). Compared with men who had never smoked, ever smokers showed an increased rate of XX and XY disomy (IRR range, 1.05–1.07; 95% CI: 1.01–1.15) and a nonsignificant increased rate of YY disomy (IRR range, 1.02–1.05; 95% CI: 0.93, 1.11) in adjusted models. Finally, in adjusted models decreased abstinence time was associated with significantly increased rates of XX (IRR range: 1.07–1.09; 95% CI: 1.01, 1.17) and XY (IRR range: 1.06–1.10; 95% CI: 1.01, 1.21) and a nonsignificant increase in total sex-chromosome disomy (IRR range: 1.01–1.09; 95% CI: 0.93, 1.20). However, there was no overall significant linear trend (data not shown).

After adjustment for age, smoking status, abstinence time, sperm concentration and motility, serum lipids, and Σ_4_PCBs, serum *p,p*´-DDE levels above the lowest quartile were associated with increased rates of XX, XY, and total sex-chromosome disomy, but not YY disomy (Table 4). For example, the highest quartile of *p,p*´-DDE (≥ 1.68 ng/g) was associated with a 60% increase in the incidence rate of XX disomy (IRR = 1.6; 95% CI: 1.4, 1.7) compared with the lowest exposure quartile (≤ 0.61 ng/g). However, the dose response appeared nonlinear, with most of the increase in disomy occurring between the first and second quartile of serum *p,p*´-DDE and without substantial additional increases across subsequent quartiles. We observed no significant relationships between exposure to *p,p*´-DDE and 1818 disomy.

**Table 4:** Crude and adjusted IRRs (95% CIs) for 1818, XX, YY, XY, and total sex-chromosome disomy by quartiles of *p,p*´-DDE for a subset of men seeking infertility evaluation, January 2000–May 2003 (*n* = 192).

*p,p*´-DDE quartile*a*				
Disomy	2nd	3rd	4th	*p*-Value for trend
1818								
Crude IRR (95% CI)		1.00 (0.90, 1.07)		1.01 (0.95, 1.09)		1.00 (0.93, 1.09)		0.44
Adjusted IRR (95% CI)*b*		0.99 (0.89, 1.08)		0.98 (0.90, 1.07)		0.98 (0.88, 1.06)		0.41
XX18								
Crude IRR (95% CI)		1.17 (1.08, 1.27)		1.27 (1.18, 1.38)		1.35 (1.24, 1.47)		< 0.001
Adjusted IRR (95% CI)*b *		1.27 (1.16, 1.38)		1.57 (1.43, 1.72)		1.57 (1.42, 1.73)		< 0.001
YY18								
Crude IRR (95% CI)		1.09 (1.01, 1.19)		1.10 (1.02, 1.20)		1.04 (0.95, 1.14)		0.31
Adjusted IRR (95% CI)*b*		1.05 (0.97, 1.15)		1.08 (0.98, 1.19)		0.95 (0.86, 1.05)		0.25
XY18								
Crude IRR (95% CI)		1.34 (1.28, 1.41)		1.19 (1.14, 1.25)		1.28 (1.22, 1.35)		< 0.001
Adjusted IRR (95% CI)*b*		1.31 (1.25, 1.38)		1.16 (1.09, 1.22)		1.30 (1.23, 1.38)		0.001
Total disomy								
Crude IRR (95% CI)		1.25 (1.21, 1.30)		1.19 (1.15, 1.24)		1.25 (1.20, 1.30)		< 0.001
Adjusted IRR (95% CI)*b*		1.25 (1.20, 1.30)		1.22 (1.17, 1.27)		1.27 (1.22, 1.33)		< 0.001
**a***p,p*´-DDE quartile cut points (ng/g serum): 0.22–0.61, 0.62–0.97, 0.98–1.67, 1.68–30.3. **b**Adjusted for age (continuous), abstinence time (three categories: ≤ 2 days, 3–4 days, ≥ 5 days), smoking (never vs. ever), log sperm concentration (continuous), log sperm motility (continuous), ∑_4_PCBs (congeners 118, 138, 153, 180; continuous), and serum lipids (continuous).								

After adjustment for age, smoking status, abstinence time, sperm concentration and motility, serum lipids, and *p,p*´-DDE, we observed an overall significant linear increase in the rate of YY, XY, and total sex-chromosome disomy for the second, third, and fourth quartiles of Σ_4_PCBs compared with the lowest quartile based on a trend test (Table 5). However, not all disomy outcomes showed significant individual quartile increases. In contrast, there was a significant decrease in the rate of XX disomy above the first quartile of Σ_4_PCBs. No significant relationships between exposure to PCBs and 1818 disomy were observed.

**Table 5:** Crude and adjusted IRRs (95% CIs) for 1818, XX, YY, XY, and total sex-chromosome disomy by quartiles of ∑_4_PCBs for a subset of men seeking fertility evaluation, January 2000–May 2003 (*n* = 192).

∑_4_PCB quartile*a*				
Disomy	2nd	3rd	4th	*p*-Value for trend
1818								
Crude IRR (95% CI)		0.99 (0.91, 1.07)		0.98 (0.92, 1.06)		1.00 (0.92, 1.10)		0.70
Adjusted IRR (95% CI)*b*		0.97 (0.90, 1.04)		0.96 (0.89, 1.05)		0.99 (0.90, 1.07)		0.67
XX18								
Crude IRR (95% CI)		0.86 (0.63, 1.19)		0.87 (0.62, 1.20)		0.96 (0.70, 1.32)		0.52
Adjusted IRR (95% CI)*b*		0.87 (0.80, 0.94)		0.68 (0.62, 0.75)		0.70 (0.63, 0.77)		< 0.001
YY18								
Crude IRR (95% CI)		1.15 (0.88, 1.51)		1.11 (0.84, 1.46)		1.26 (0.96, 1.66)		< 0.001
Adjusted IRR (95% CI)*b*		1.09 (0.99, 1.19)		0.99 (0.90, 1.10)		1.13 (1.02, 1.26)		0.02
XY18								
Crude IRR (95% CI)		1.31 (1.02, 1.68)		1.07 (0.82, 1.40)		1.28 (0.98, 1.65)		< 0.001
Adjusted IRR (95% CI)*b*		1.35 (1.28, 1.42)		1.10 (1.04, 1.17)		1.34 (1.27, 1.43)		< 0.001
Total disomy								
Crude IRR (95% CI)		1.16 (0.94, 1.45)		1.03 (0.82, 1.29)		1.19 (0.95, 1.49)		< 0.001
Adjusted IRR (95% CI)*b*		1.17 (1.13, 1.22)		0.98 (0.93, 1.02)		1.13 (1.08, 1.19)		< 0.001
∑_4_PCBs includes congeners 118, 138, 153, and 180. **a**∑_4_PCB quartile cut points (ng/g serum): 0.16–0.29, 0.30–0.53, 0.54–0.80, 0.81–6.14. **b**Adjusted for age (continuous), abstinence time (three categories: ≤ 2 days, 3–4 days, ≥ 5 days), smoking (never vs. ever), log sperm concentration (continuous), log sperm motility (continuous), *p,p*´-DDE (continuous), and serum lipids (continuous).								

In secondary analyses of alternative groupings of PCB congeners, after adjustment for age, smoking status, abstinence time, sperm concentration and motility, serum lipids, and *p,p*´-DDE, there was a significant increase in the rate of XY and total sex-chromosome disomy for the second, third, and fourth quartiles compared with the lowest quartile for Σestrogenic PCBs and Σdioxin-like PCBs and an increase in YY disomy for Σestrogenic PCBs. However, we observed a significant decrease in XX disomy for higher quartiles of Σestrogenic and Σdioxin-like PCBs (data not shown).

We conducted sensitivity analyses for all disomy outcomes after excluding three men who had total sex-chromosome disomy rates that far exceeded current published ranges (total sex-chromosome disomy > 5%); these men were excluded to prevent undue statistical influence from these extreme values. In the reanalysis, the results remained essentially unchanged (data not shown). We also conducted a sensitivity analysis after excluding eight men with total nuclei scored < 1,000, as disomy estimates can be impacted by too few nuclei scored. Again, in the reanalysis, the results remained essentially unchanged (data not shown).

## Discussion

This is the first epidemiologic study to investigate the relationship between environmental exposure to *p,p*´-DDE and PCBs with sperm sex-chromosome disomy. Our results suggest a significant increase in the rate of XX, XY, and total sex-chromosome disomy for the second, third, and fourth quartiles of of *p,p*´-DDE compared with the lowest quartile; these results persisted even after adjustment for potential confounders. For Σ_4_PCBs, we observed a significant increase in the rate of YY, XY, and total sex-chromosome disomy after adjustment for potential confounders. In contrast, the rate of XX disomy was significantly decreased above the first quartile of Σ_4_PCBs. We observed no significant relationships between PCBs or *p,p*´-DDE and 1818 disomy.

Most of the observed increase in disomy occurred between the first and second quartiles of serum PCB and *p,p*´-DDE exposures, with approximately constant increases across subsequent exposure quartiles. A saturation dose–response relationship has been observed in other studies of organochlorine health effects, including a recent study of organochlorines and male peripubertal growth (Burns et al. 2011). Furthermore, men in the first exposure quartile were not significantly different from men in higher exposure quartiles (e.g., with regard to demographic, lifestyle, or semen parameters; data not shown), so population differences across exposure groups are unlikely to explain the apparent threshold effect.

Because no prior studies have investigated organochlorine exposure and sperm aneuploidy, comparisons of these findings with other studies are limited. Six epidemiologic studies have evaluated pesticide exposure and sperm aneuploidy (reviewed by Perry 2008); however, none have evaluated DDT or *p,p*´-DDE exposure, nor has an epidemiologic study evaluated PCB exposures and sperm aneuploidy. Of the four pesticide studies that investigated aneuploidy in the sex chromosomes (Recio et al. 2001; Smith et al. 2004; Xia et al. 2004, 2005), significant increased associations were seen with organophosphate metabolites (Recio et al. 2001), ambient air fenvalerate levels (Xia et al. 2004), and ambient air carbaryl levels (Xia et al. 2005). These compounds are fast acting and have half-lives that range from days to weeks. One of the four pesticide studies did not find an association between insecticide, fungicide, or herbicide exposures and sex-chromosome diploidy (Smith et al. 2004). However, exposures in that study were determined by self-report and job exposure ratings and not from biomonitoring or environmental air monitoring.

Aneuploidy is most likely a consequence of disruption of meiosis during gametogenesis. It is unclear how hormone-disrupting compounds such as PCBs and *p,p*´-DDE might interfere with the meiotic process. In studies of meiosis, Martin (2006) and Sun et al. (2008) have demonstrated that infertile men have impaired chromosome synapsis, a significantly decreased frequency of recombination, and an increased frequency of chromosomes that are missing a recombination site. Such errors make these cells susceptible to meiotic arrest and production of aneuploid gametes. Altered recombination has an effect on nondisjunction (Ferguson et al. 2007; Shi and Martin 2001). Susceptibility of these chromosomes to nondisjunction may be due to reduced connections among homologous pairs in nonrecombinant chromosomes. A few toxicological studies have linked some pesticides to errors in somatic cell recombination (Giri et al. 2002; McCarroll et al. 2002), and chemicals known to disrupt hormone signaling may interfere with recombination sequences. In rodent models, changes in the endocrinologic environment of the testes have been shown to affect the rate of meiotic segregation errors (Mroz et al. 1999; Oppedisano et al. 2002). Alterations in endocrine homeostasis induced by organochlorines could thereby adversely affect meiosis. However, identifying the likely mechanism(s) for an epidemiologic association between PCBs and *p,p*´-DDE with sex-chromosome disomy will require further investigation via experimental animal and *in vitro* studies.

It is also unclear how increased exposure to PCBs might be protective for XX while increased exposure to *p,p*´-DDE appeared to increase XX disomy. Because sex-chromosome disomy is a rare event, it is susceptible to measurement error, which could decrease the precision of the effect estimate. However, the error would be nondifferential and therefore not bias the results. Importantly, XY disomy was the most common disomy type and showed the most consistent relationship with exposures. XX and YY occurred less frequently than did XY. Less-consistent exposure associations with XX or YY may, in part, reflect even greater measurement error for these events.

Alternatively, in a study exploring the Y:X sperm ratio in men exposed to PCBs and *p,p*´-DDE, Tiido et al. (2006) showed an association between increased exposures to organochlorines and increased ejaculated Y-bearing sperm. Tiido et al. (2005) hypothesized that the decrease in X-bearing ejaculated sperm may be due to the effects of organochlorines on the formation of X-chromosome micronuclei, which are chromosomal fragments or whole chromosomes that are not incorporated into daughter nuclei because of chromosomal breakage or dysfunction (Gauthier et al. 1999). A recent study by Pedersen et al. (2010) showed a positive association between dioxin-like PCBs and increased micronuclei frequencies in cord blood of infants exposed *in utero*. PCB 118, a dioxin-like PCB congener that is a partial aryl hydrocarbon receptor agonist, was associated with an increase micronuclei frequency in somatic cells of Belgian adults environmentally exposed to PCBs (De Coster et al. 2008). Although no studies have explored the impacts of micronucleation on meiotic cells (i.e., germ cells), increased X-chromosome micronucleation, hypothesized to cause a decrease in X-bearing ejaculated sperm, might lead to decreased XX disomy. In addition, it is unclear whether exposure to PCBs versus *p,p*´-DDE and other organochlorines differentially impact micronuclei formation in germ cells, specifically sex chromosomes. Confirmation of these findings in other populations is needed.

The men in this study were members of subfertile couples seeking infertility evaluation. Although they may differ from men in the general population, there is currently no evidence showing that they would differ in ways that would alter their response to PCBs or *p,p*´-DDE. Thus, our results may apply to general population samples as well. Participants were heterogeneous in their semen profiles, and more that half had normal semen parameters. The heterogeneity of men attending fertility centers should increase the generalizability findings from a clinic-based study population, but replication in other populations is needed to confirm the generalizability of our findings.

Chromosome disomy frequency estimates from the present study (mean ± SD: XX, 0.41 ± 0.40; YY, 0.38 ± 0.33; XY, 1.11 ± 0.83) were higher than previously reported ranges among men spanning the continuum of semen abnormalities using manual cell counting (XX, 0.03–0.37; YY, 0.04–0.21; XY, 0.06–0.42) (Egozcue et al. 1997; Templado et al. 2005). In a study of fertility clinic patients who were known reciprocal translocation carriers, XY disomy was as high as 4.1% (Moretti et al. 2009). Because we did not routinely perform karyotypes, we do not know the prevalence of underlying reproductive conditions such as Robertsonian translocations in our clinic sample. Additionally, in a recent study of sperm disomy among andrology clinic patients using automated methods, Tempest et al. (2010) also reported higher total sex-chromosome disomy (averaging between 1.3 and 1.5% total disomy) compared with previously reported ranges (Egozcue et al. 1997). However, they found no differences between manual and automated estimates.

Importantly, our previous validation studies comparing automated and manual results for sex-chromosome disomy in a small sample of normozoospermic men from a fertility clinic also found higher disomy estimates than previously reported in men spanning the semen parameter continuum (Egozcue et al. 1997; Templado et al. 2005), but results did not differ between manual and semiautomated methods (Perry et al. 2007, 2011). Taken together, the use of a validated semiautomated method for counting disomic sperm was considered a strength in that study because it allowed for objective processing of a large number of samples, which is important for etiologic hypothesis testing.

## Conclusion

The results of the present study suggest that men with higher serum *p,p*´-DDE levels have significant increases in the rates of XX, XY, and total sex-chromosome disomy. Men with higher serum levels of Σ_4_PCBs had significant increases in the rates of YY, XY, and total sex-chromosome disomy. In contrast, we observed a significant decrease in the rate of XX disomy for higher serum levels of Σ_4_PCBs. It remains unclear why exposure associations differ among the various sex-chromosome disomy outcomes. This is the first epidemiologic study to investigate the relationship between environmental exposure to *p,p*´-DDE and PCBs and sperm sex chromosome disomy. Therefore, these findings require replication in other populations.
